# A Comprehensive Specimen-Specific Multiscale Data Set for Anatomical and Mechanical Characterization of the Tibiofemoral Joint

**DOI:** 10.1371/journal.pone.0138226

**Published:** 2015-09-18

**Authors:** Snehal Chokhandre, Robb Colbrunn, Craig Bennetts, Ahmet Erdemir

**Affiliations:** 1 Computational Biomodeling (CoBi) Core, Lerner Research Institute, Cleveland Clinic, Cleveland, Ohio, 44195, United States of America; 2 Department of Biomedical Engineering, Lerner Research Institute, Cleveland Clinic, Cleveland, Ohio, 44195, United States of America; 3 BioRobotics and Mechanical Testing Core, Lerner Research Institute, Cleveland Clinic, Cleveland, Ohio, 44195, United States of America; Tel Aviv University, ISRAEL

## Abstract

Understanding of tibiofemoral joint mechanics at multiple spatial scales is essential for developing effective preventive measures and treatments for both pathology and injury management. Currently, there is a distinct lack of specimen-specific biomechanical data at multiple spatial scales, e.g., joint, tissue, and cell scales. Comprehensive multiscale data may improve the understanding of the relationship between biomechanical and anatomical markers across various scales. Furthermore, specimen-specific multiscale data for the tibiofemoral joint may assist development and validation of specimen-specific computational models that may be useful for more thorough analyses of the biomechanical behavior of the joint. This study describes an aggregation of procedures for acquisition of multiscale anatomical and biomechanical data for the tibiofemoral joint. Magnetic resonance imaging was used to acquire anatomical morphology at the joint scale. A robotic testing system was used to quantify joint level biomechanical response under various loading scenarios. Tissue level material properties were obtained from the same specimen for the femoral and tibial articular cartilage, medial and lateral menisci, anterior and posterior cruciate ligaments, and medial and lateral collateral ligaments. Histology data were also obtained for all tissue types to measure specimen-specific cell scale information, e.g., cellular distribution. This study is the first of its kind to establish a comprehensive multiscale data set for a musculoskeletal joint and the presented data collection approach can be used as a general template to guide acquisition of specimen-specific comprehensive multiscale data for musculoskeletal joints.

## Introduction

Biomechanics of the knee is dependent on the anatomy and mechanical properties of its numerous tissue structures as well as the interactions between them. Passive tissue structures of the knee include ligaments, menisci, and articular cartilage, which have unique structural organization at a lower spatial scale, reflecting each tissue type's specialized mechanical role [1,2]. Multiscale anatomical and mechanical characterizations of the knee at joint, tissue and cell scales are important to better understand both healthy and diseased joint function, and to develop preventative measures and treatment strategies for diseased states and injury.

Past experimentation on knee biomechanics can be classified as: (1) those exploring the functional response of the joint and (2) those aiming to identify the anatomical and mechanical properties of the joint or its underlying tissues. The presence of functional loads makes *in vivo* experimentation useful for understanding joint behavior under physiological conditions [3–5]. However, *in vivo* tissue mechanical behavior is challenging to study and often not possible. *In vitro* studies have explored the functional response of the joint, to evaluate biomechanical interventions such as surgical techniques and to determine their influence on joint mechanics [6]. Nonetheless, a primary focus of *in vitro* experimentation has been for the mechanical characterization of the joint or its underlying tissues [7,8], but not necessarily both. For example, at the joint level, the kinematics-kinetics response of the knee has been well characterized [9], but was not supported by subsequent tissue characterization. Similarly, many studies have characterized individual tissue mechanics [10–12], but not necessarily elaborate on the role of variations in tissue properties on overall joint response. There is a distinct lack of specimen-specific information encompassing multiple scales. While the plethora of data separately available in literature, for joint response and for tissue response, can be aggregated, due to large variations in reported population data (dimensions, geometry, properties, etc.) [1,10], it is challenging to use this information to infer biomechanical relationships between different spatial scales and even within the same scale, among different tissue structures.

A specimen-specific data set, acquired at both the joint and tissue levels, may provide the opportunity to relate tissue scale anatomical and mechanical properties to cell level anatomical organization and to overall biomechanical function of the joint. Such capacity may also find utility to understand knee mechanics in diseased states. For example, a multiscale comparison between healthy and pathological knees can differentiate at what level the overall joint mechanics change, by examining if such changes are a result of the disease's impact on the anatomical or material properties of one or more substructures, or due to an alteration on how they interact.

Another potential utility of a multiscale specimen-specific data set is in modeling & simulation. Predictive and descriptive studies of the mechanical function of the knee and its substructures commonly employ computational approaches, in particular finite element (FE) analyses. Such studies require anatomical and mechanical data at single or multiple scales for development of models. It is also critical that FE models closely represent the behavior of the joint of interest, both anatomically and mechanically. This is essential if these models are intended to be used to support clinical decision making (in surgery, for rehabilitation, or to test implant performance) or for the purpose of personalized medicine [13]. Studies employing FE analysis often use specimen-specific geometry, but currently do not use corresponding specimen-specific tissue properties; tissue properties are commonly obtained from prior literature [14,15]. The extent of joint level mechanical validation may also be limited, with a few exceptions [16] One should note that, a model may provide reasonable predictions of kinematics-kinetics response when compared against the mean and standard deviation of the sample population but may significantly deviate from specimen-specific response, particularly when large variability exists. Role of such variability has been previously evaluated in FE studies. For example, Dhaher et al. [17] performed a sensitivity analysis showing that uncertainties in ligament material properties significantly affect FE model predictions.

Thus, the goals of this study were two-fold. First, this work aimed to establish a multiscale experimentation protocol to acquire anatomical and mechanical data from a given knee, including the joint, tissue, and cell levels. Second, this study targeted to provide a multiscale data set for a given tibiofemoral joint, which can, in future, guide explorations of biomechanical relationships between spatial scales and development of a computational model of the tibiofemoral joint informed by specimen-specific anatomy and mechanics.

## Methods

**Fig 1:**
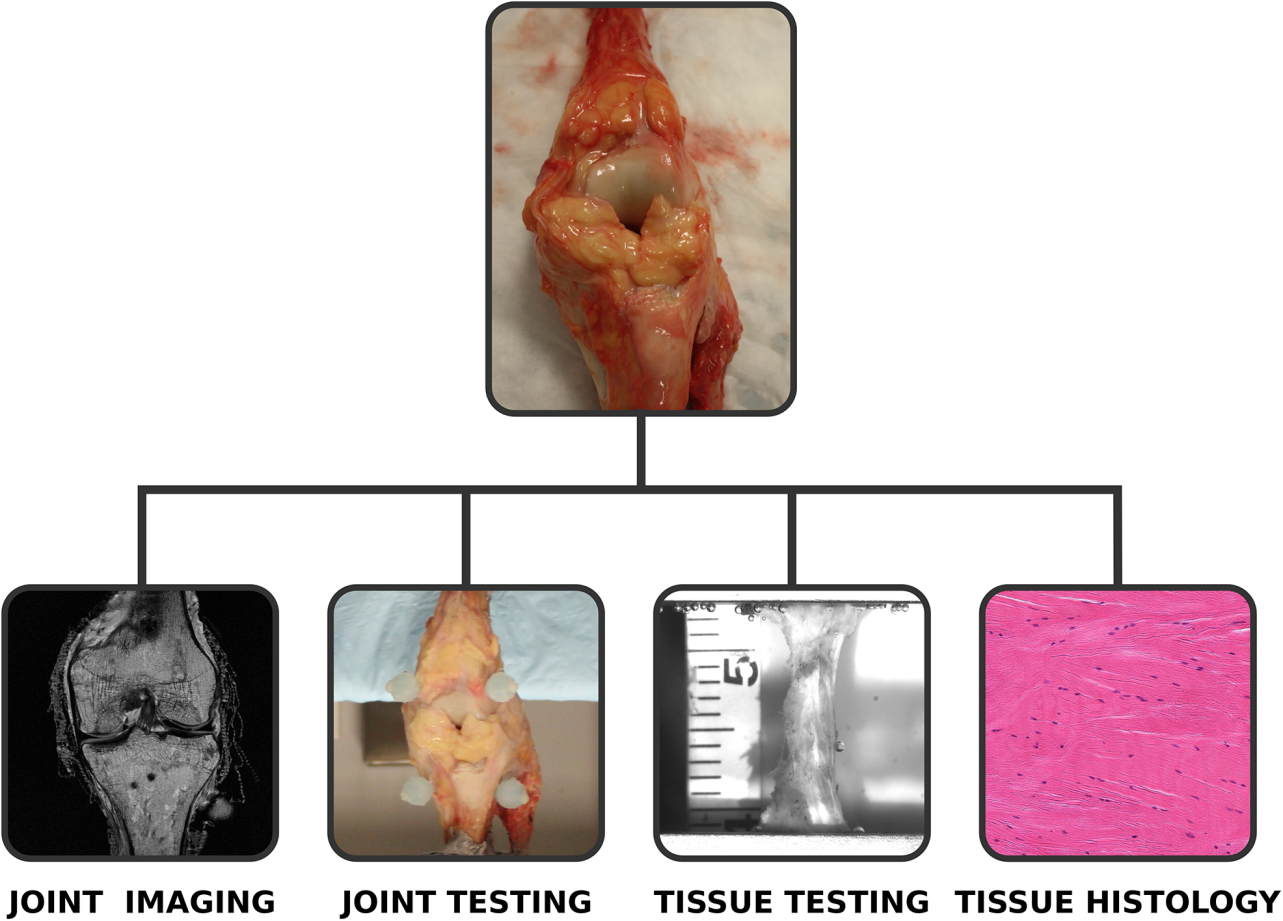
Overview of multiscale data acquisition on the tibiofemoral joint. Data collection includes magnetic resonance imaging (to reconstruct joint anatomy), joint mechanical testing (to interpret the mechanical behavior of the joint), tissue mechanical testing (to understand tissue material properties), and histology (to evaluate cell level and microstructural information).

Multiscale anatomical imaging and mechanical testing were conducted on a single knee specimen (Fig 1). Scale-specific experimentation protocols are detailed in following sections. Some of the methodological information have been previously described in abridged forms in [18] and [19].

### Specimen Characteristics, Preparation, and Registration

Data collection was performed on a left cadaver knee. The specimen was procured from LifeLegacy Foundation (Tucson, AZ). No IRB approval was acquired as the donor information was de-identified by the supplier before the specimen was sent to the investigators. The donor was a 34 years old female with a normal body mass index (BMI) of 19 and no known knee or foot injuries, surgeries, osteoarthritis or inflammatory arthritis. The specimen was prepared by an orthopedic surgeon so that only the salient passive structures of the tibiofemoral joint remained intact, including the femur, tibia, femoral and tibial articular cartilage, anterior and posterior cruciate ligaments, medial and lateral collateral ligaments, and menisci. To match the image and mechanical testing coordinate systems, registration marker sets (consisting of three 10 mm radius hollow plastic spheres filled with water-based ultrasound gel) were fixed to each of the femur and tibia using plastic screws. In a preliminary analysis, this particular method of registration was found to have a relative accuracy within 2% for measuring the distance between two markers. Twelve points on these markers were manually digitized in the images collected during anatomical imaging. During joint mechanical testing, following the mounting of the specimen on the mechanical testing setup, a three-dimensional digitizer (MicroScribe G2L, Immersion Corp., San Jose, CA) was used to capture 12 points on the outer surface of each spherical marker. Spheres were fit to each set of points on a given marker from both imaging data and experimental three-dimensional digitizer data, locating fiducial points at the marker centers, to allow future alignment of the imaging/model coordinate system with the joint testing coordinate system.

### Anatomical Imaging

For imaging, the specimen was secured to a non-magnetic holder designed to keep the joint in a neutral state while also preventing any relative movement of the joint. Magnetic resonance (MR) images were acquired at the University Hospitals & Case Western Reserve University, Cleveland, OH, using a 4 Tesla scanner (Medspec, Bruker Biospin Corp., Billerica, MA) and the following imaging protocol: T1 Turbo Spin Echo, without fat suppression, having an in-plane resolution of 0.3125 mm, and 1.5 mm slice thickness. Image sets were obtained in all three orthogonal planes (coronal,axial and sagittal) using the same protocol. In addition to the various tissues of interest, the imaging protocol also allowed clear delineation of registration markers for future digitization from the MR images in order to establish the relationship between mechanical testing and imaging coordinate systems.

### Joint Mechanical Testing

Mechanical testing was conducted by securing the knee joint to a custom testing apparatus that was mounted on a six degrees of freedom motion control robot (Rotopod R-2000, Parallel Robotic Systems Corp., Hampton, NH, USA) with an added actuator to provide a seventh degree of freedom to allow large flexion angles [20]. A spatial load transducer (SI-2500-400, ATI Industrial Automation, Apex, NC) recorded joint kinetics (three forces, three moments, resolution: 0.5 N and 0.7 Nm in the x and y dimensions (anterior-posterior and medial-lateral directions) and 1.1 N and 0.7 Nm in the z dimension (superior-inferior direction). A software framework, simVITRO, developed in LabVIEW™ (version 8.2, National Instruments, Austin, TX), was used to control the robot and measure the resultant kinematics. The kinetics and kinematics measurements were sampled at 100 Hz. Using a three-dimensional digitizer (MicroScribe G2L, Immersion Corp., San Jose, CA), a joint coordinate system was established between the femur and tibia and related to the coordinate systems of the load cell, robot, and registration markers. The loads were measured in the tibia fixed coordinate system and the kinematics were measured in the joint coordinate system as described by Grood and Suntay [21]. Single axis laxity tests and combined loading tests were performed for flexion angles ranging from 0° to 90° in 30° increments. Laxity tests were conducted at each flexion angle using the following loading conditions: (1) internal-external rotation moments from 0 to ± 5 Nm in increments of 1 Nm; (2) varus-valgus moments from 0 to ± 10 Nm in increments of 2.5 Nm; (3) anterior-posterior forces from 0 to ± 100 N in increments of 10 N. Loads were controlled to the target set-points, and off-axis loads were minimized during these test conditions using the simVITRO real time force feedback controller. A quasi static combined loading test consisted of internal-external rotation moments ranging from 0 to ±5 Nm and varus-valgus moments ranging from 0 to ± 10 Nm while under an anterior or a posterior drawer force of 100 N. All laxity tests were performed at 30° flexion at the end of mechanical testing to assess the repeatability of the procedure and to rule out any potential damage to the specimen. The testing protocol was adapted from Borotikar [22], with the exception that higher flexion angles were incorporated in the protocol.

### Tissue Mechanical Testing

**Fig 2:**
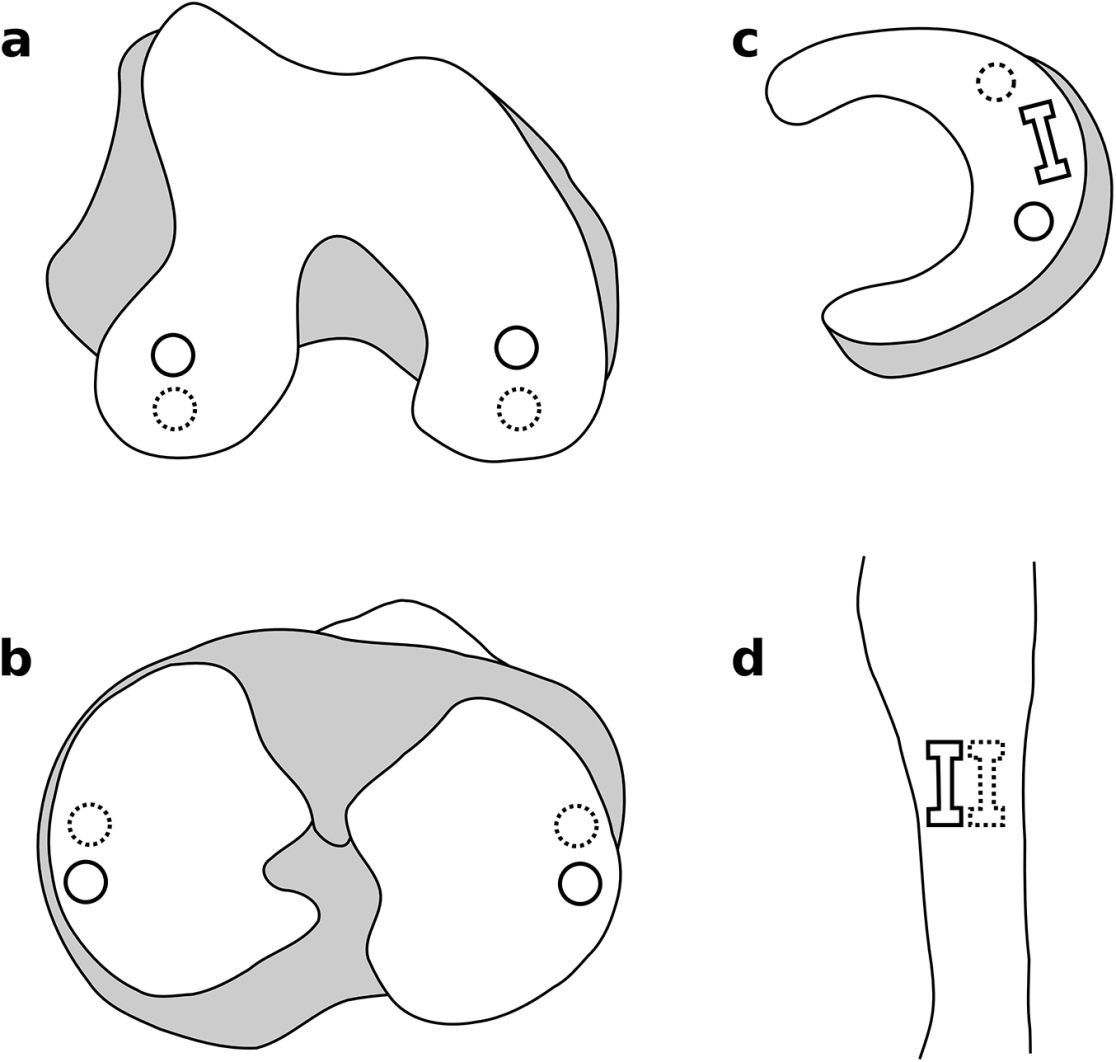
Tissue sample locations. a) & b) Confined compression samples of cartilage from the medial and lateral femoral condyles and tibial plateaus. c) Confined compression and tensile test samples from the menisci. d) Tensile test samples from the mid-substance region of the ligaments. Solid lines represent mechanical testing samples; dashed lines represent histology samples.

Following joint testing, substructures of the tibiofemoral joint were dissected and all the tissues of interest were isolated. Overall twelve samples were mechanically tested from the femoral and tibial articular cartilage, cruciate and collateral ligaments, and the menisci. Full thickness cartilage samples were harvested from the medial and lateral femoral condyles and medial and lateral tibial plateaus using a 5mm diameter cylindrical punch (Fig 2). These samples were tested under confined compression. Similar compression samples were prepared for the medial and lateral menisci (Fig 2). Meniscus samples were also tested in uniaxial tension. For this purpose, 5mm by 1 mm (test dimension) punch was used to prepare dumbbell shaped samples. These samples were harvested from the deeper circumferential region of both menisci. Ligaments were tested under uniaxial tension after punching dumbbell shaped samples, 10 mm by 2 mm, from all four ligaments. These samples were harvested from the mid-substance region of the ligaments (Fig 2). A vibratome (Leica VT1200 S, Leica Microsystems Inc., Buffalo Grove, IL) was used to prepare uniform thickness samples for all the cartilage and meniscus tests. Sample thicknesses for all tensile samples were measured before each test using a constant-pressure (~0.001 MPa) linear variable displacement transducer (LVDT) probe. Three thickness measurements were taken for each sample and the average values are reported. Special fixtures and clamps were utilized depending on the nature of the tests. In case of tensile tests, sand paper and tissue adhesive were used along with serrated metal clamps to prevent the test samples from slipping during mechanical testing. The compression samples were tested in a specially designed confined compression chamber. A 5 μm pore size sintered stainless steel filter was placed on the exposed articular surface and the opposing surface was placed flush to the nonporous bottom of the testing chamber. All the tests were conducted on an Instron 5543 Series testing system (Instron, Canton, MA) using a 50 N load cell (accuracy:± 0.25% full scale, Honeywell Sensotec, Columbus, Ohio). Displacement was controlled using MTS Flextest SE controllers (MTS, Eden Prairie, MN). All the specimens were immersed in a saline bath and kept at 37°C during testing. Freeze-thaw cycles during sample acquisition and preparation were kept minimal; all tissue samples went through a maximum of three freeze-thaw cycles, including those required before and after anatomical imaging and joint testing.

All the tests incorporated stress-relaxation conditions. Each cartilage and meniscus sample was tested at 5%, 10% and 15% strain with 45 min hold time after each strain; a ramp loading at a strain rate of 20%/s was applied to reach the target strains for both confined compression and uniaxial tension. A pre-load of 0.05N was applied before the stress-relaxation tests to obtain the initial test length or thickness of the cartilage and meniscus samples. In the case of the ligaments, a pre-load of 0.1 N was applied to obtain the initial test length, followed by 10 preconditioning cycles at an amplitude of 0.25 mm. A stress-relaxation test was performed at 5% and 10% strain at a strain rate of 20%/s for all the ligament samples with 60 min hold time after each strain application. For all testing, the data acquisition frequency for ramp loading was 170 Hz and for the hold cycle, it was 30 Hz. Video data were also recorded during the tensile tests using an Imperx IPX-VGA210L camera system (resolution: 640 x 480; Imperx, Inc., Boca Raton, FL). Tissue marker ink was used to place markers on the tensile test samples. The camera system was synchronized with the data acquisition system and video data were captured at 50 Hz for ramp loading and 2 Hz for hold cycle during all tensile tests.

Moduli of the linear region (aggregate moduli in the case of confined compression), for instantaneous loading and at the relaxed state, were obtained for all samples to allow comparisons with literature. The slope of the stress-strain response between two highest strain levels, as prescribed by the experimentation, was used to estimate the modulus. For calculation of the instantaneous modulus, peak values of stress at the end of ramp loading were utilized. For calculation of the modulus at the relaxed state, final data points at the relaxation cycles were used. Stresses were calculated by normalizing the applied force by the cross-sectional area of the sample. Strains for tensile tests represented grip-to-grip strain.

### Histology

Cellular organization in tissue samples were characterized through histology, following hematoxylene & eosin (H&E) staining and microscopy. For the cartilage and ligaments, histology samples were harvested from the region neighboring the mechanical test sample locations. For meniscus, radial blocks were cut that retained entire thickness of the tissue. These samples were first fixed in formalin for 8–10 days, which was followed by washing the samples in phosphate-buffered saline (PBS). The samples were dehydrated and embedded in paraffin before being cut. One 10 μm thick slice was obtained for each tissue type along the depth and stained with H&E to highlight the cell distribution and fiber structure. To characterize the cell populations, a histology image obtained at a resolution of 0.74 μm/pixel was analyzed using a 100 x 100 μm kernel size to quantify cell density. This quantification was done for all histology samples. Cellular information was obtained from the histology data for all the tissues. Raster-scanning through each image using a 100x100μm kernel, the kernel coordinates (upper-left corner), grid area, tissue area, nuclei count, nuclei coordinates (object’s centroid), and mean nuclei distance were obtained for all images.

## Results

### Imaging and Joint Mechanical Testing

**Fig 3:**
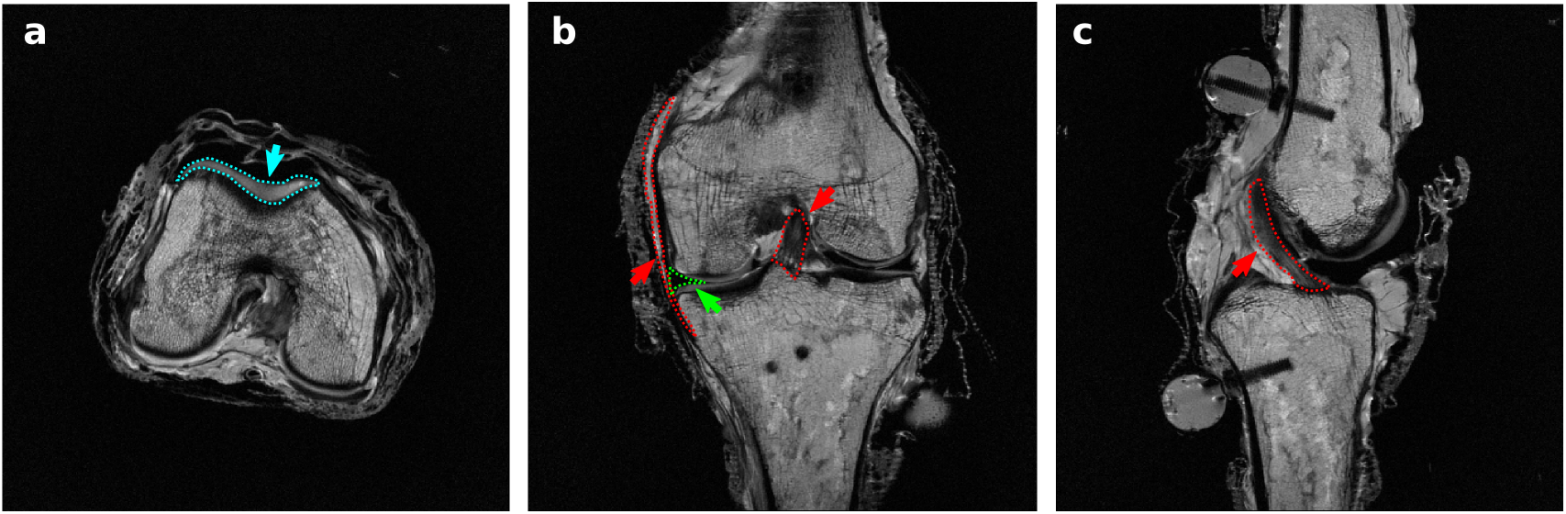
Magnetic resonance images of the tibiofemoral joint. a) Axial section; cartilage is highlighted. b) Coronal section; anterior cruciate ligament, medial collateral ligament, and medial meniscus are highlighted. c) Sagittal section; anterior cruciate ligament is highlighted.

**Fig 4:**
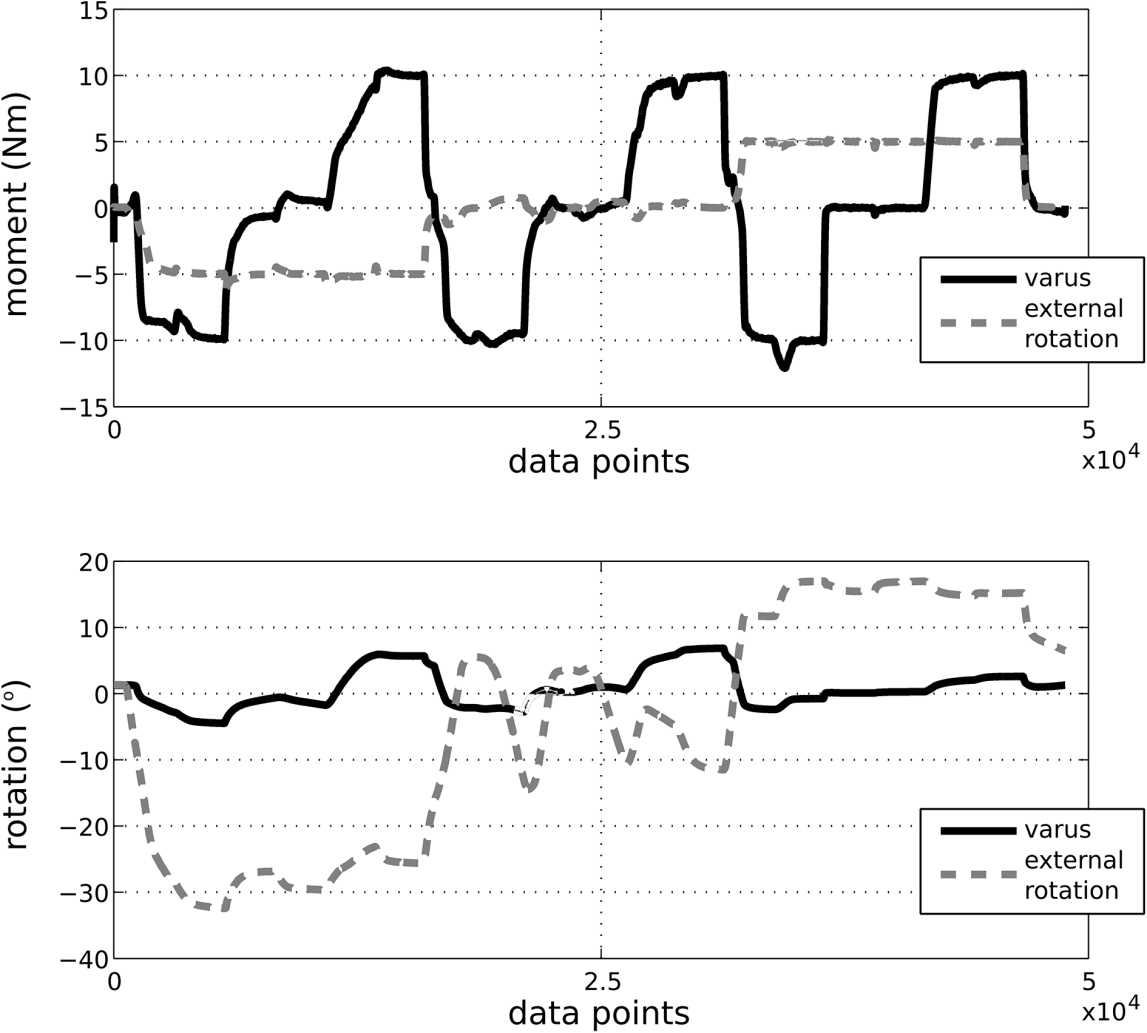
Time history of applied varus and external rotation moments and resulting joint rotations during combined loading. Tibiofemoral flexion was set at 30° during this particular test. The loads are represented in the tibia fixed coordinate system; movements are described in the anatomical joint coordinate system.

**Fig 5:**
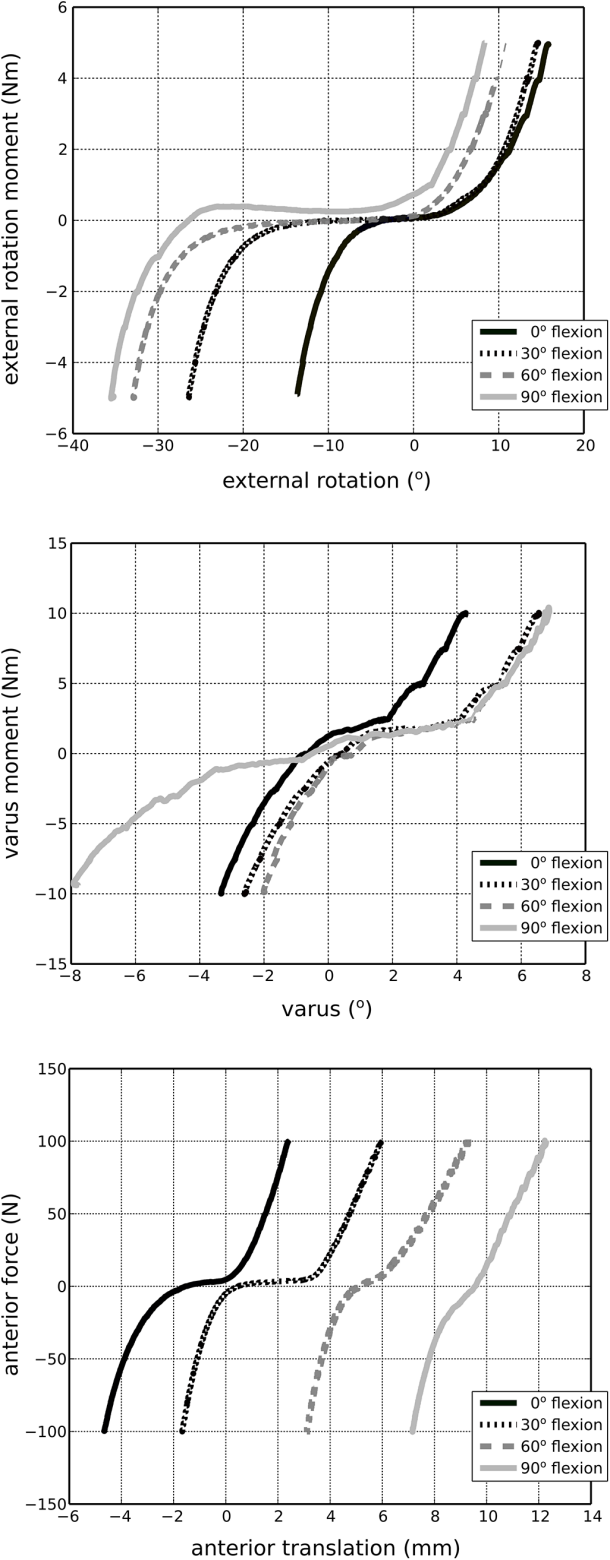
Joint kinematics-kinetics response in dominant axes for various laxity tests. The loads are represented in the tibia fixed coordinate system; movements are described in the anatomical joint coordinate system.

**Fig 6:**
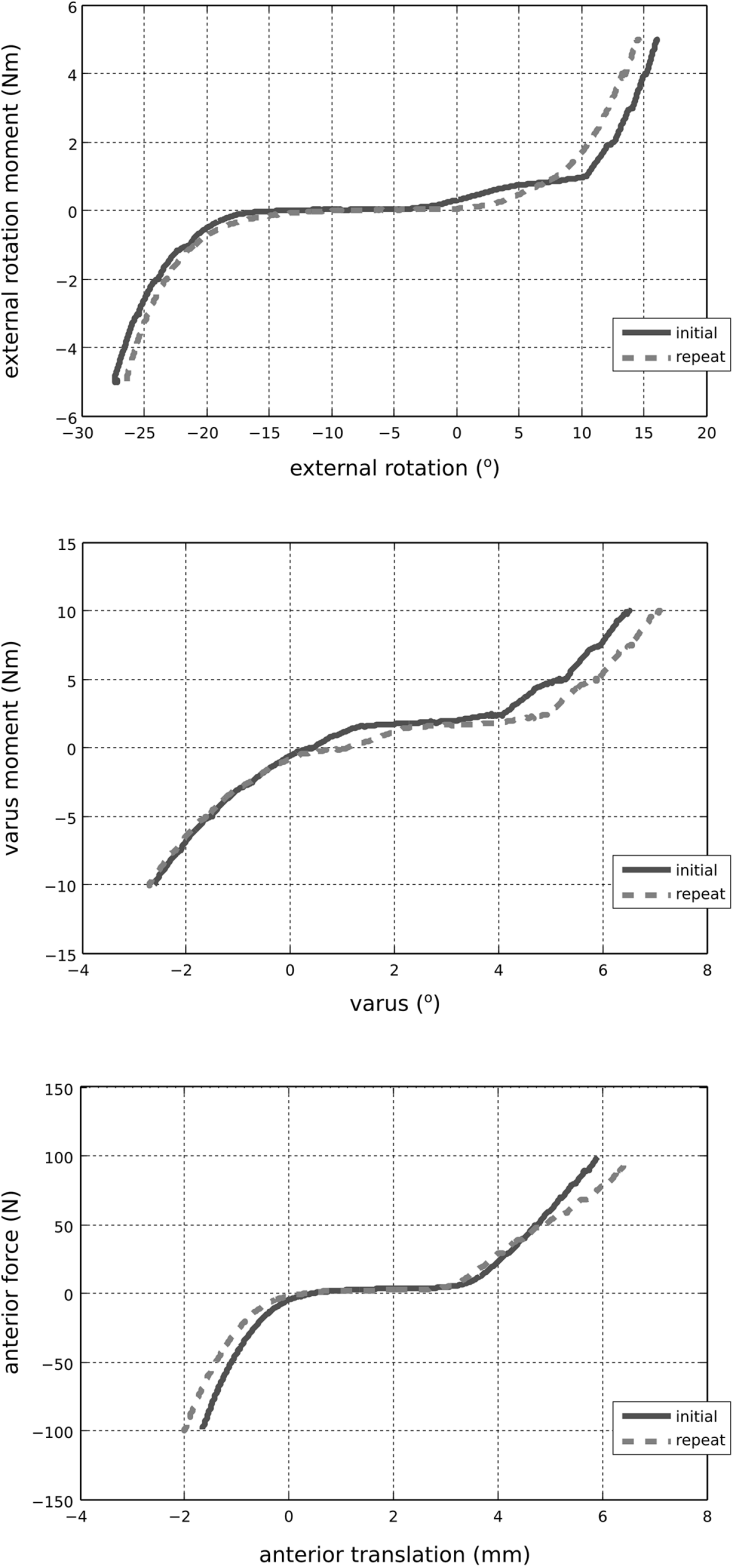
Reproducibility of joint kinematics-kinetics response in dominant axes for laxity tests at 30° flexion. The loads are represented in the tibia fixed coordinate system; movements are described in the anatomical joint coordinate system. Repetition of these laxity tests, after completion of all desired mechanical testing on the joint, indicated that the specimen remained intact during testing.

The MR images provided detailed anatomical information for the boundaries of soft tissues and bones (Fig 3). A sample time history of mechanical joint testing including the prescribed rotational kinetics and the resultant kinematics in a combined loading scenario is shown in Fig 4. Fig 5 summarizes kinematics-kinetics relationships for dominant axes during laxity testing. For example, at a 30° flexion angle, the range of translational motion of the joint at ±100 N anterior drawer force was approximately -2 to 4 mm and at 90° flexion angle the range of varus-valgus motion was approximately -8° to 6° for ±10 Nm varus-valgus moments. Increased range of internal-external rotational and varus-valgus laxities were observed with increasing flexion angle, whereas the range of anterior-posterior laxity decreased slightly at higher flexion angle. The knee remained intact, as illustrated by the similarities in joint kinematics-kinetics response for repeated laxity tests at 30° flexion angle (Fig 6).

### Tissue Mechanical Testing

**Fig 7:**
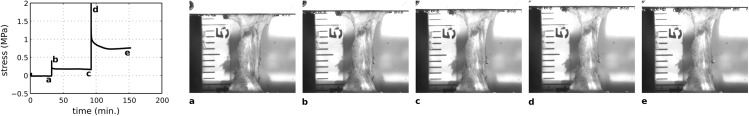
Stress-relaxation response of the anterior cruciate ligament. Tensile stress data is shown along with the visualization of the sample at the beginning and end of the ramp and hold cycles.

**Fig 8:**
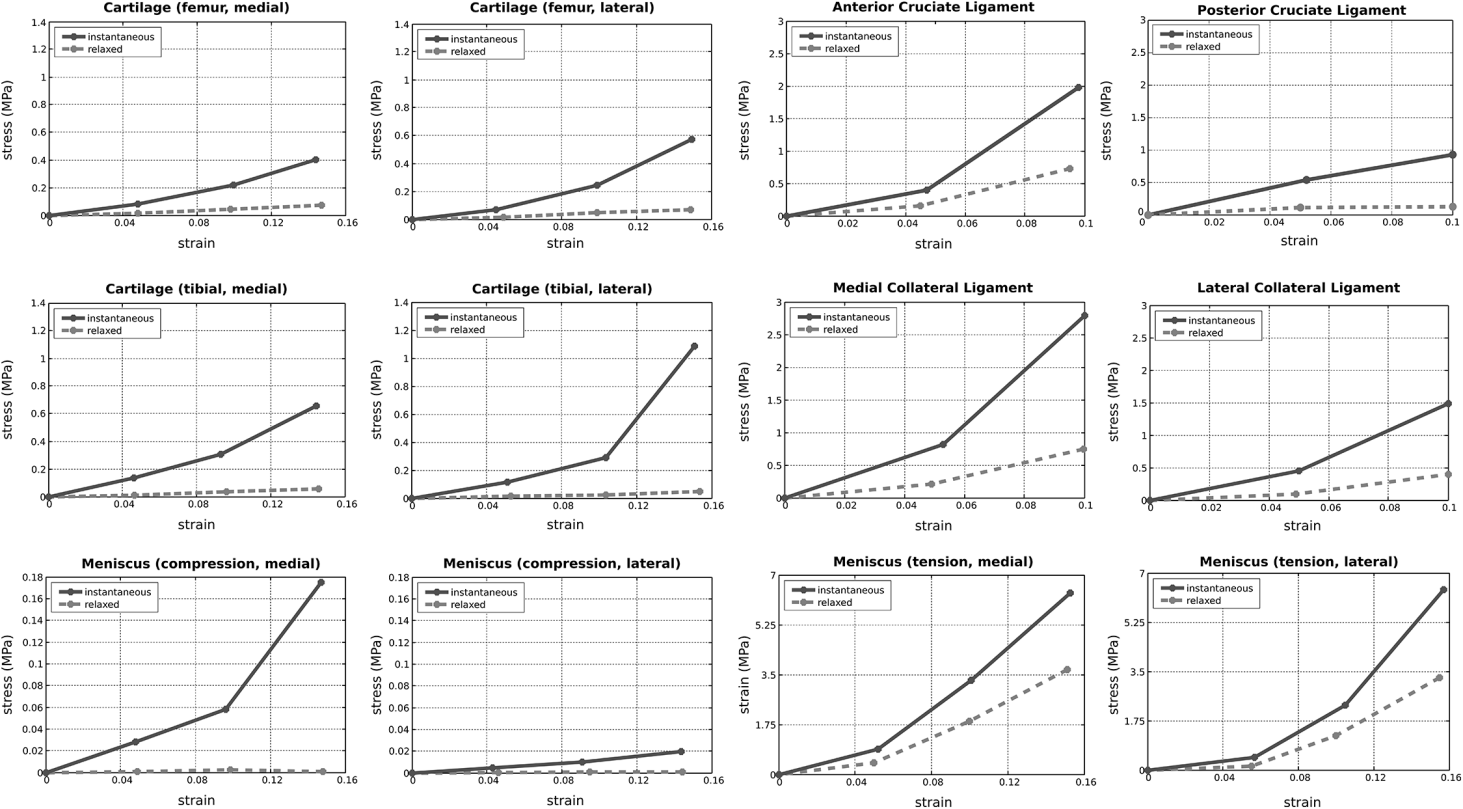
Reconstructed stress-strain response of all tissues at target strain levels, for instantaneous and relaxed states. Nominal stresses and grip-to-grip strains are reported. For estimates of moduli, refer to Table 2. The posterior cruciate ligament failed during the second ramp loading (10% strain); this describes the unexpected decrease in the stiffness of this tissue for larger strains. At the relaxed state, the stress response of the menisci during compression was low; the values reported may potentially be hindered by experimentation capacity.

Tissue sample locations along with sample dimensions are listed in Table 1. As an example, the stress-relaxation response of the anterior cruciate ligament under tension is provided in Fig 7. A summary of stress-strain behavior of all the specimens, as calculated from data points at the end of ramp loading (instantaneous) and hold cycles (relaxed), can be found in Fig 8. Estimated moduli for the linear region of the instantaneous and relaxed stress-strain responses are also provided in Table 2.

**Table 1:** Details of the locations and dimensions for all samples for mechanical tissue testing.

**Sample**	**Location**	**Testing Type**	*Diameter* (mm)		*Thickness* (mm)
Cartilage	Medial femoral condyle	Confined compression	5		2.24
Cartilage	Lateral femoral condyle	Confined compression	5		2.19
Cartilage	Medial tibial plateau	Confined compression	5		2.34
Cartilage	Lateral tibial plateau	Confined compression	5		2.41
Meniscus	Medial	Confined compression	5		2.32
Meniscus	Lateral	Confined compression	5		2.04
**Sample**	**Location**	**Testing Type**	*Width* (mm)	*Length* (mm)	*Thickness* (mm)
Meniscus	Medial	Uniaxial tension	1	4.46	0.53
Meniscus	Lateral	Uniaxial tension	1	5.30	0.70
ACL	Mid-substance	Uniaxial tension	2	12.86	1.58
PCL	Mid-substance	Uniaxial tension	2	13.30	0.90
LCL	Mid-substance	Uniaxial tension	2	12.75	1.39
MCL	Mid-substance	Uniaxial tension	2	13.02	1.29

Diameter was assumed from punch diameter. Width was assumed from punch width, length was estimated from grip-to-grip distance at pre-load; and thickness was measured using a probe. ACL: anterior cruciate ligament; PCL; posterior cruciate ligament; MCL: medical collateral ligament; LCL: lateral collateral ligament.

**Table 2:** Moduli of all tissue samples for the linear region of the stress-strain response, for instantaneous and relaxed states.

Sample	Location	Testing Type	Relaxed Modulus (MPa)	Instantaneous Modulus (MPa)
Cartilage	Medial femoral condyle	Confined compression	0.61	4.04
Cartilage	Lateral femoral condyle	Confined compression	0.48	6.48
Cartilage	Medial tibial plateau	Confined compression	0.42	6.73
Cartilage	Lateral tibial plateau	Confined compression	0.54	16.90
Meniscus	Medial	Confined compression	(-0.036)	2.28
Meniscus	Lateral	Confined compression	(0.00)	0.198
Meniscus	Medial	Uniaxial tension	36.44	61.16
Meniscus	Lateral	Uniaxial tension	41.4	82.04
ACL	Mid-substance	Uniaxial tension	11.44	33.98
PCL	Mid-substance	Uniaxial tension	0.34	8.13
MCL	Mid-substance	Uniaxial tension	10.47	42.04
LCL	Mid-substance	Uniaxial tension	6.20	20.76

Moduli were approximated by calculating the slope of stress-strain data reported for the last two target strain levels prescribed during the experiments, see Fig 8. For confined compression samples, aggregate moduli are reported. The posterior cruciate ligament failed during the second ramp loading (10% strain); this describes the low values for the moduli of this tissue. At the relaxed state, the stress response of the menisci during compression was low; the values reported may potentially be hindered by experimentation capacity (shown in parenthesis). ACL: anterior cruciate ligament; PCL; posterior cruciate ligament; MCL: medical collateral ligament; LCL: lateral collateral ligament.

### Histology

**Fig 9:**
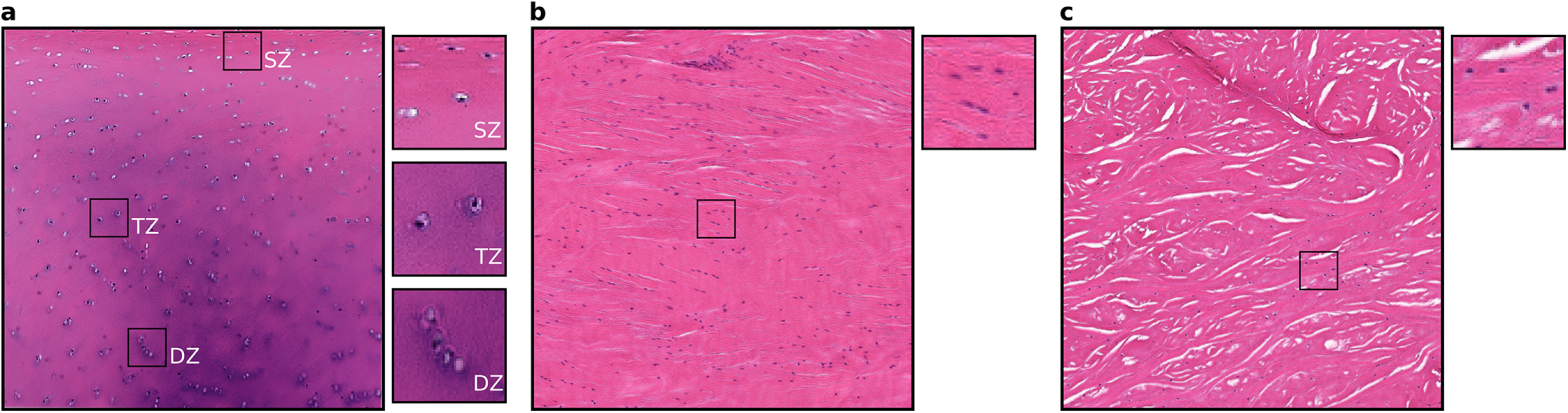
Histology sections for various tissues of the tibiofemoral joint. a) Femoral medial articular cartilage with sections from superficial (SZ), transitional (TZ) and deep (DZ) zones illustrating zonal variations in cell distributions. b) Lateral collateral ligament. c) Medial meniscus. The large sections are approximately 1 mm x 1 mm and the small sections are approximately 100 μm x 100 μm.

Overall, ten histology images were obtained for the three tissue types. Histology images (as shown in Fig 9) provide information on cellular organization, which may be useful in building specimen-specific cell scale computational models. For instance, the average number of cells in 300 μm x 300 μm regions (aggregated from 100 μm x 100 μm kernels), arbitrarily selected from the superficial, transitional and deep zones of articular cartilage sections of the medial femoral condyle were found to be 14, 9 and 5, respectively. Similarly, the number of cells for arbitrarily selected 300 μm x 300 μm regions in the anterior cruciate ligament and lateral meniscus were found to be 24 and 11, respectively.

## Discussion

The goals of this study were to aggregate joint and tissue testing protocols for multiscale anatomical and mechanical data acquisition for the knee joint and to obtain and summarize a multiscale mechanical testing and anatomical imaging data set for a tibiofemoral joint and its underlying tissue structures. It is anticipated that aggregation of these can be utilized for relational characterization of joint and tissue anatomy and mechanics in the future. This data set can also be used to develop a specimen-specific finite element representation of the tibiofemoral joint at multiple resolutions.

The multiscale data set acquired from one knee joint was summarized to illustrate the breadth of specimen-specific information. Mechanical properties were briefly compared against literature to evaluate the sample knee's joint and tissue level mechanical characteristics in relation to the previously reported population data. From the perspective of joint and tissue anatomy, the MR images were adequate to delineate the geometry of the various tissues (Fig 3). More specialized imaging protocols may be used in the future targeting specific tissues. Imaging protocols employed by studies such as those done by Peterfy et al. [23], may be used as guidelines to acquire separate image sets specific to cartilage, ligaments, or meniscus.

The overall joint mechanical response in terms of range of motion and trends under laxity loading was in agreement with the literature, albeit acknowledging the differences in coordinate system definitions and loading scenarios in our study and those of others [9,24–27]. As the flexion angle increased, the zero load state of the joint appeared to have translated anteriorly. We did not subtract the kinematic offset for each degree of freedom at each flexion angle tested, rather raw data are shown. As a result, movement of the zero load position as a function of flexion angle was expected (Fig 5 top and bottom). Some of this movement may have been physiological but may as well have been influenced by our coordinate system definition. The variability in coordinate system definitions can result in discrepancies when comparing kinematics results across multiple specimens, whether *in silico*, *in vitro*, or *in vivo* [28]. Our raw data also indicated a large varus-valgus laxity at 90° flexion when compared to other flexion angles (Fig 5, middle). While this may be attributed to coordinate system related issues described above, it may well be representative of the knee tested in this study. These discrepancies may also be due to the loading control scheme adapted during robotics testing; which may introduce uncertainties, particularly for minimization of off-axes loads.

Moduli values were obtained to evaluate material properties of the tissues for instantaneous loading and at relaxed states during confined compression and uniaxial tensile tests. The aggregate moduli of the cartilage at the relaxed state were comparable to values reported in the literature [29–31]. The tensile moduli of the meniscal samples were comparable to previously reported values [32]. At a relaxed state, menisci exhibited low stresses during confined compression and our calculated aggregate moduli for menisci was low and sometimes non-physiological (Table 2). This may be attributed to our experimentation capacity inducing uncertainties in measurement of low forces, as in meniscus testing, possibly due to resolution and experiment control. In addition, on a visual inspection, the menisci appeared to be degenerated and the lower compressive moduli may be a reflection of the tissue state. An interesting note is that the lateral femoral and tibial cartilage samples appeared to be stiffer than their medial counterparts whereas, the opposite was noted for the meniscal samples. This may be an indication of a negative correlation between the behavior of cartilage and menisci under compression, which may warrant further investigation. A similar relationship between tensile properties of the bovine cartilage and meniscus was also noted in a recent study by Danso et al. [33].

Ligament moduli for this specimen, irrespective of the ligament type, were found to be lower than those reported in the literature [34]. For example, while the stress-strain behavior of the medical collateral ligament for our specimen is similar to non-linear behavior reported elsewhere [35], the modulus is lower [12]. The posterior cruciate ligament appeared to have been damaged during the test (in the second ramp loading stage), which is evident from the stress-strain behavior (Fig 8). Some unanticipated low decreases in tissue loading were observed in the raw data of some tensile relaxation tests, e.g., of the medical and lateral collateral ligaments, and medial and lateral menisci. Slippage at the grips is a known challenge in tensile tests and we suspected that this may have been the cause of a decreased load reading. However, no obvious or visible slippage or tissue failure was noticed from video data.

Compared to previous histological images of cartilage, ligament and meniscus, our histology images indicated similar fibrous architecture and cellular distributions [36–38]. Based on the study done by Hunziker et al [39], the number of cells per 300 μm x 300 μm x 10 μm were expected to be approximately 21, 9 and 6 for superficial, transitional and deep zones in the cartilage, respectively. Our data showed 14, 9 and 5, respectively. Cell counts reported in the study by Hunziker et al. [39] were acquired using stereological methods [40], whereas the number of cells reported in our study did not accommodate this. Regardless, the geometry information at the cell level, including cellular organization and potentially fiber orientation, will be useful in building physiologically realistic and specimen-specific cell scale FE models. However, as only one slice was imaged for each sample, the histological samples may not be entirely representative of the whole tissue.

With the established aggregate of protocols and guidelines, studies benefiting from both anatomical and mechanical specimen-specific information can be conducted. For instance, these combined testing protocols can be used to explore biomechanical properties at different spatial scales for the same specimen. This level of specimen-specific data, when and if acquired for a large number of specimens, may be useful in exploring and establishing relationships between multi-level mechanical markers (joint and tissue, joint and cell) of healthy and pathological joints. For example, it will be possible to understand the effects of tissue level changes on overall joint mechanics. Our study, even with one specimen, implies potential hypotheses and research directions related to relative properties of meniscus and cartilage in medial and lateral compartments, i.e., do medial compartment have different tissue properties than lateral side? If so, why? Will the meniscus develop as more compliant if the cartilage is stiffer? How will this influence overall joint response? Such questions motivate and require additional specimen-specific characterization at multiple scales and our multiscale experimentation specifications can be used to acquire such data for such studies.

In future, availability of a multiscale data set also provides the possibility to explore the required level of specimen-specificity in computational representations of the tibiofemoral joint for predictive assessment via simulations of healthy subjects/specimens and for various pathologies or injuries. Comprehensive development and evaluation of computational models require data across multiple spatial scales. However, specimen-specific models are generally limited to only the anatomical representation, e.g., [41]. Validation studies are also usually limited to comparisons against literature, for a limited number of load cases, e.g., [42], or against other specimens or sample populations, e.g., [15]. A recent study by Kiapour et al. [15] built a detailed FE model of the knee and confirmed its predictive capacity against joint mechanics response from a sample population of cadaver knees. They noted the potential limitations due to lack of specimen-specific tissue properties, also elaborating on the technical challenges related to acquisition of such data and developing a specimen-specific model. Song et al. [43] attributed the sensitivity of ACL stresses in the FE model to material properties and to the fact that the validation data used in the study was not for the same specimen on which the geometry was based built. Yao et al. [44] found that the properties of cartilage and meniscus affect meniscal motion in FE analysis predictions. Many numerous investigations including the aforementioned studies can significantly benefit from the availability of specimen-specific data at all spatial scales, including anatomy and mechanical properties.

To demonstrate the potential role of specimen-specific information for modeling purposes, a simple model of ACL stiffness was constructed. Stiffness was calculated as a function of ACL geometry and material properties, *EA*/*L*, where *E* is the elastic modulus, *A* is the cross-sectional area and L is the slack length of the tissue. Specimen-specific geometry was estimated from three-dimensional reconstruction of the ACL from MR images: *A* = 59.96 mm^2^; *L* = 33.78 mm. Stiffness was predicted twice, first by using average ACL material property (modulus) reported in literature (average of 10 specimens in a study by Chandrashekhar et al [45]; *E* = 99 MPa), then by using specimen-specific ACL material property measured in our study (*E* = 33.98 MPa). The axial stiffness values obtained using the two moduli values were found to be 60.32 N/mm (using specimen-specific information) and 175.76 N/mm (using information from literature). This analysis indicated that predicted ACL stiffness relying on literature based material properties may largely deviate from the value relying on complete specimen-specific information. Such a modeling workflow is common in finite element analysis, e.g., geometry is acquired from subject/specimen anatomy whereas material properties are assumed to be the same as average properties reported in literature.

Prospective multiscale modeling & simulation studies relying on our data are also anticipated. Joint, tissue and cell scale information obtained in this study can be utilized for explorations of the mechanical load sharing pathway of the tibiofemoral joint [46], this time in a specimen-specific manner. For example, tissue scale response obtained from specimen-specific joint level simulations can be boundary conditions for specimen-specific cell level models (including cell shapes and density) to predict cellular level mechanical response.

Some limitations remain in regard to the general utility of the data set. Data acquisition was conducted on only one specimen. Some tissue components, which may be of interest for other purposes, such as the patella, were removed prior to joint testing. In addition, the number of samples from each tissue was limited. Also, zonal and regional properties were not obtained for the tissues. This may limit the information required to represent the comprehensive material behavior particularly for micro-scale models of the tissue. Specimen-specific microstructural-level mechanical tests were also not performed. Further, improvements in tissue-specific imaging protocols may result in the acquisition of enhanced anatomical information. Mechanical tissue testing protocols may need to be improved to address technical issues such as sample size measurements. Thickness values for tensile specimens were measured by a contact based system which may underestimate the sample thickness, therefore the cross-sectional area. This may result in uncertainties in stress calculations and therefore identification of the material properties for tissues. It may be also be possible to acquire data in a more detailed manner, e.g., larger number of target strain levels during stress-relaxation tests.

Despite varying degrees of limitations, this study provides the framework for a more complete understanding of the mechanics of the tibiofemoral joint, with a prospective utility for the development of multiscale computational representations founded on specimen-specific data. The entire data set is freely and openly accessible to the community at large, in particular for those who may not otherwise have the resources to acquire such comprehensive information, to foster advances in studies of the tibiofemoral joint: https://simtk.org/home/j2c/- "Multiscale data set (version 1.0.0)” in the Downloads section.
